# Randomized phase II study with two gemcitabine- and docetaxel-based combinations as first-line chemotherapy for metastatic non-small cell lung cancer

**DOI:** 10.1186/1479-5876-6-65

**Published:** 2008-10-31

**Authors:** Alessandro Passardi, Lorenzo Cecconetto, Monia Dall'Agata, Claudio Dazzi, Enzo Pasquini, Giovanni Oliverio, Federica Zumaglini, Wainer Zoli, Oriana Nanni, Carlo Milandri, Giovanni Luca Frassineti, Dino Amadori

**Affiliations:** 1 Istituto Scientifico Romagnolo per lo Studio e la Cura dei Tumori, Meldola, Italy; 2 Istituto Oncologico Romagnolo, Forlì, Italy; 3 Department of Oncology, Santa Maria delle Croci Hospital, Ravenna, Italy; 4 Department of Oncology, Infermi Hospital, Rimini, Italy

## Abstract

**Background:**

Docetaxel and gemcitabine combinations have proven active for the treatment of non-small cell lung cancer (NSCLC). The aim of the present study was to evaluate and compare two treatment schedules, one based on our own preclinical data and the other selected from the literature.

**Methods:**

Patients with stage IV NSCLC and at least one bidimensionally-measurable lesion were eligible. Adequate bone marrow reserve, normal hepatic and renal function, and an ECOG performance status of 0 to 2 were required. No prior chemotherapy was permitted. Patients were randomized to arm A (docetaxel 70 mg/m^2^on day 1 and gemcitabine 900 mg/m^2 ^on days 3–8, every 3 weeks) or B (gemcitabine 900 mg/m2 on days 1 and 8, and docetaxel 70 mg/m2 on day 8, every 3 weeks).

**Results:**

The objective response rate was 20% (95% CI:10.0–35.9) and 18% (95% CI:8.6–33.9) in arms A and B, respectively. Disease control rates were very similar (54% in arm A and 53% in arm B). No differences were noted in median survival (32 vs. 33 weeks) or 1-year survival (33% vs. 35%). Toxicity was mild in both treatment arms.

**Conclusion:**

Our results highlighted acceptable activity and survival outcomes for both experimental and empirical schedules as first-line treatment of NSCLC, suggesting the potential usefulness of drug sequencing based on preclinical models.

**Trial registration number:**

IOR 162 02

## Background

Lung cancer remains the leading cause of cancer-related mortality in the western world. Non-small cell lung cancer (NSCLC) accounts for approximately 80% of these thoracic malignancies, with 1.2 million new cases diagnosed worldwide each year [1]. The vast majority of NSCLC patients present with advanced, inoperable disease and are, therefore, candidates for palliative chemotherapy. The role of chemotherapy as an integral part of the treatment of lung cancer has grown significantly, especially in the last few years. In metastatic disease it prolongs survival and improves quality of life in patients with good performance status, and also appears to provide symptomatic improvement in patients with decreased performance status [2,3].

Among active chemotherapeutic agents, cisplatin has historically been considered the most effective in both the palliative treatment of metastatic disease and the combined-modality therapy of locally advanced disease. In stage IV NSCLC, cisplatin-based chemotherapy results in improved survival with respect to supportive care alone. In an analysis of more than 2,000 patients with advanced NSCLC treated in Southwest Oncology Group (SWOG) trials, cisplatin emerged as an independent variable of improved survival.

Over the last decade, a number of new chemotherapeutic agents that are active in NSCLC have undergone clinical development, such as the taxanes, irinotecan, vinorelbine and gemcitabine. Phase II trials of these new agents in combination with platinum have shown impressive response and survival results, leading to their widespread clinical application. Subsequent randomized trials comparing these novel regimens with cisplatin alone or with "older" platinum combinations have generally confirmed a therapeutic advantage for the new agent-platinum schedules, albeit with a lesser improvement in survival than that indicated by earlier phase II studies [4-7].

Although in the recent past several randomized studies have compared these new doublets, no important differences have emerged, and so all tested doublets can be considered as reasonable choices for patients with advanced NSCLC [8-10]. Recent randomized trials have also compared the efficacy of platinum-free and platinum-based regimens, showing equivalence [11-14]. However, platinum-based polychemotherapy remains the standard treatment for metastatic disease.

We evaluated the preclinical activity of docetaxel and gemcitabine in 2 established NSCLC cell lines (RAL, CAEP) [15]. The sequence docetaxel→gemcitabine produced only a weak synergistic interaction in RAL but a strong synergism in CAEP cells. The synergistic interaction increased in both cell lines after a 48-hour washout between drug administrations. Conversely, simultaneous treatment induced an antagonistic effect in both cell lines and the sequential scheme gemcitabine→docetaxel produced a weak synergistic effect only in RAL cells. The synergistic activity of docetaxel→48-hour washout→gemcitabine was confirmed in 11 out of 14 primary cultures. We also investigated the activity of 2 administrations of gemcitabine after docetaxel in NSCLC cell lines and found that a 48-hour washout between the 2 administrations of gemcitabine resulted in a stronger cytotoxic activity than that obtained with a 72-hour washout [16].

On the basis of the data obtained from our previous phase I clinical study of advanced NSCLC in which escalating doses of both drugs (docetaxel 50–70 mg/m^2 ^on day 1, gemcitabine 800–1200 mg/m^2 ^on days 3–8, every 21 days) were used, we defined the optimal dose-treatment: docetaxel 70 mg/m^2 ^on day 1 and gemcitabine 900 mg/m^2 ^on days 3–8 [17,18].

In the present randomized phase II trial, we evaluated the activity of this new treatment schedule together with an empirical schedule selected from the literature: gemcitabine 900 mg/m^2 ^on days 1 and 8 and docetaxel 70 mg/m^2 ^on day 8, every 3 weeks.

## Materials and methods

### Eligibility Criteria

Patients with histologically and/or cytologically confirmed stage IV NSCLC; age ≥ 18 years; Eastern Co-operative Oncology Group (ECOG) Performance Status 0–2; life expectancy > 12 weeks; adequate hepatic and renal function: creatinine and total bilirubin ≤ 1.5 × upper limit of normal, AST and ALT ≤ 3 × upper limit of normal (≤ 5 in patients with liver metastases); adequate bone marrow reserve: absolute neutrophil count ≥ 1.5 × 10^3^/L, platelet count ≥ 100 × 10^3^/L, hemoglobin ≥ 9 g/dl. Patients were required to have at least one lesion that was bidimensionally measurable according to WHO criteria.

### Exclusion Criteria

Active serious infections or severe concomitant diseases (at the discretion of the investigator); known central nervous system tumors, including metastatic brain disease; pregnancy or breast-feeding; previous or concurrent malignancy other than lung cancer, with the exception of non melanoma skin cancer or curatively treated carcinoma *in situ *of the uterine cervix; previous chemotherapy in an adjuvant setting or for metastatic disease. No other anticancer treatments, with the exception of bisphosphonates and palliative radiotherapy of non target lesions, were allowed.

All patients gave written informed consent to receive treatment and the study was examined and approved by the Ethics Committee of the Local Health and Social Services of each participating center, in accordance with the ethical standards laid down in the 1964 Declaration of Helsinki.

### Treatment Plan

Patients who fulfilled all inclusion and exclusion criteria were randomized to receive the experimental regimen (arm A), defined in the phase I trial as docetaxel 70 mg/m^2 ^on day 1 and gemcitabine 900 mg/m^2 ^on days 3–8, or the empirical regimen (arm B), consisting of gemcitabine 900 mg/m^2 ^on days 1 and 8, and docetaxel 70 mg/m^2 ^on day 8. Both schedules were repeated every 21 days and administered on an outpatient basis.

Patients received antiemetics and granulocyte colony-stimulating factor at the physicians' discretion. Palliative and supportive treatment for tumor-related symptoms was administered as required. All patients received intravenous (i.v.) or intramuscular (i.m.) dexamethasone 8 mg bid 24 hours before each infusion of docetaxel up to a total of six doses.

Treatment was given for a maximum of 8 cycles and was discontinued in cases of unacceptable toxicity, disease progression, patient refusal or when, in the judgement of the investigator, a different treatment would be more appropriate for the patient's overall clinical status.

Dose reductions were made in the presence of hematological toxicity (ANC < 0.5 × 10^9^/L and/or platelet count < 50 × 10^9^/L lasting more than 7 days) or grade 3 non hematological toxicity (other than alopecia and nausea/vomiting). Patients were taken off study if grade IV febrile neutropenia, thrombocytopenia with severe bleeding, or any grade IV non hematological toxicity occurred.

### Statistical Plan

The primary endpoint of the study was to assess the overall response rate (ORR) in patients treated in each separately analyzed arm. Secondary endpoints were toxicity, duration of response, time to progression (TTP) and overall survival (OS).

This randomized phase II trial can be considered as two simultaneous phase II studies: the sample size for each arm was calculated using Simon's one-stage design with a 5% α error and 10% β error. Assuming a poor ORR P0 = 10% and an acceptable objective response rate P1 = 30%, it was planned to recruit 33 patients. If the response number was ≥ 7 in each arm, the combination would be considered active and warrant further investigation.

Efficacy and toxicity analyses were performed on all patients who received at least one dose of study treatment. Statistical analysis included simple descriptive statistics.

ORRs were calculated and 95% confidence intervals (95% CI) were derived from the exact binomial distribution. Kaplan-Meyer estimations were used to evaluate response duration, progression-free survival (PFS) and OS. No formal statistical test was performed. Block balanced randomization lists were performed for each center.

### Evaluation of Activity and Toxicity

Screening evaluation included full medical history, physical and neurological examinations, tumor measurements (by CT and bone scans), cardiac function examination (ECG), hematological and biochemistry analyses, and other evaluations if clinically indicated.

Treatment activity was assessed every two months according to WHO criteria. A complete response (CR) was defined as the disappearance of all lesions and no appearance of new disease for at least 4 weeks. Partial response (PR) was defined as a reduction by at least 50% in the sum of the products of the two longest diameters of all lesions, maintained for at least 4 weeks with no appearance of new disease. CR + PR was rated as the overall response rate. Stable disease (SD) was defined as a less than 50% reduction or less than 25% increase in the sum of the products of the two perpendicular diameters of all measured lesions, with no appearance of new disease. Progressive disease (PD) was an increase of more than 25% in the size of target lesions, or the appearance of an unequivocal new lesion.

Toxicity was evaluated according to National Cancer Institute Common Toxicity Criteria version 2.0 after each treatment cycle. Hematochemical assays testing hematological, liver and renal toxicity were performed on days 1, 8 and 15 of each cycle.

Response duration was defined as the interval between the dates of first documented CR, or study entry in the case of PR, and first documented sign of disease progression. PFS and TTP were measured from the date of study entry until the date of disease progression or death, and survival was measured from the date of study entry until the date of death from any cause.

The actual cumulative dose for each drug was calculated and patients were classified into 4 groups (< 50%, 50–69%, 70–84%, > 85%) on the basis of the percentage of the actual cumulative dose with respect to the planned cumulative dose.

Similarly, relative dose intensity (RDI) was defined as the total cumulative dose over time (if a patient received chemotherapy according to the protocol without any dose reduction or delay, RDI = 100%). The RDI for each drug was calculated and patients were classified into 4 groups (< 50%, 50–69%, 70–84%, > 85%) on the basis of the percentage of the RDI with respect to the planned dose intensity.

## Results

### Patient Characteristics

Between November 2001 and December 2005, a total of 81 patients with first-line stage IV NSCLC were recruited from the Medical Oncology Departments of Forlì, Rimini and Ravenna hospitals (Istituto Oncologico Romagnolo group). Baseline patient demographics are summarized in Table 1. The two treatment groups were well balanced for gender, age, performance status, disease stage, and histology. Of the 81 enrolled patients, four did not receive study treatment because of ineligibility (1 patient had severe renal dysfunction and 3 patients withdrew consent).

**Table 1:** Patient and disease characteristics at baseline

	**Arm A (n = 40)**		**Arm B (n = 41)**	
	n	(%)	n	(%)
**Received treatment**	39	(97.5)	38	(92.7)
**Age, years**				
Median (range)	63 (35–77)		63 (48–74)	
**Gender**				
Male	33	(82.5)	32	(78.0)
Female	7	(17.5)	9	(22.0)
**ECOG performance status**				
0	19	(47.5)	20	(48.8)
1	16	(40)	16	(39)
2	4	(10)	3	(7.3)
Missing	1	(2.5)	2	(4.9)
**Histological classification**				
Adenocarcinoma	22	(55)	23	(56.1)
Epidermoidal carcinoma	14	(35)	12	(29.3)
Other NSCLC	4	(10)	6	(14.6)
**Site of disease**				
Lung ± lymph nodes	14	(35)	14	(34.1)
Lung and 1 metastatic site	12	(30)	15	(36.6)
Lung and ≥ 2 metastatic sites	9	(22.5)	10	(24.4)
Extra-pulmonary disease	5	(12.5)	2	(4.9)
**Previous surgery for neoplastic disease**				
Yes	4	(10)	5	(13)
*Palliative*	*4*		*2*	
*Curative*	*0*		*3*	
No	35	(90)	33	(87)

### Treatment Activity

Of the 77 treated patients, 69 (89.6%) were assessable for tumor response (35 in arm A, 34 in arm B): one patient withdrew consent after the first treatment cycle, five were not assessable for response because of treatment discontinuation due to severe toxicity during the first cycle, and two were taken off study before evaluation because of serious adverse events, which were not considered treatment-related.

Tumor response rates were 20% (95% CI 10.0 – 35.9) in arm A and 18% (95% CI 8.6 – 33.9) in arm B (Table 2). Disease control, i.e. CR, PR, or stable disease, was achieved in 54% of arm A patients and 53% of arm B patients.

**Table 2:** Best tumor response

	**Arm A**		**Arm B**	
	**n**	**(%)**	**n**	**(%)**
**Partial response**	7	(20)	6	(18)
**No change**	12	(34)	12	(35)
**Progressive disease**	16	(46)	16	(47)

**Figure 1:**
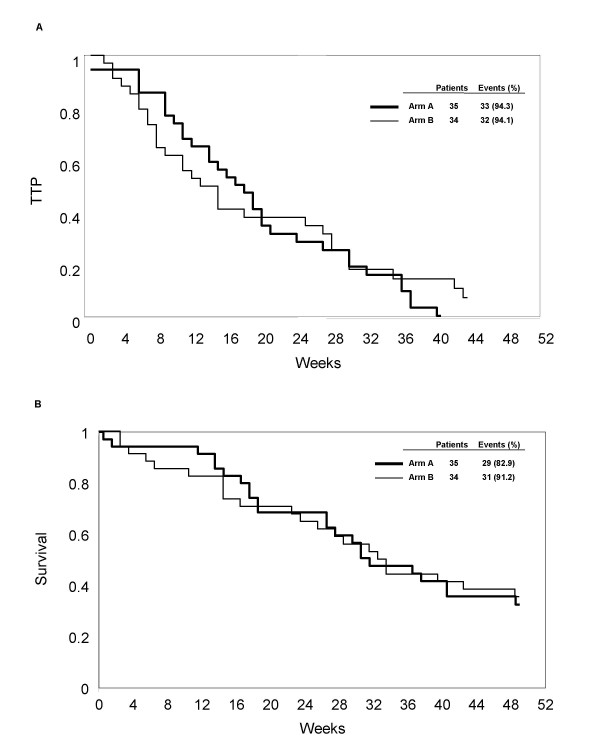
Time to progression (TTP) (A) and overall survival (OS) (B).

The median response duration was 20 weeks (range 14 – 36) and 30 weeks (range 27 – 105) in arms A and B, respectively. The median TTP in arm A was 18 weeks (95% CI 12 – 21) and 14 weeks (95% CI 8 – 27) in arm B (Figure 1A). Median survival in arms A and B was 32 weeks (95% CI 27 – 49) and 33 weeks (95% CI 24 – 54), and 1-year survival was 33% (95% CI 17 – 48) and 35% (95% CI 19 – 51), respectively (Figure 1B).

### Treatment Safety and Toxicity

A total of 292 cycles (149, arm A; 143, arm B) were administered during the study, with a median of 3 cycles per patient (range 1–8 cycles) (Table 3). With regard to the actual cumulative dose in arm A, 85% of patients received > 85% of docetaxel, while the remaining 15% received a cumulative dose of more than 70% of that planned. In arm B, 74% of patients received > 85% of the planned dose, 18% received between 50 and 84%, and only 8% were administered < 50% of the planned dose.

**Table 3:** Treatments administered

	**Arm A**	**Arm B**
**Total number of cycles**	**149**	**148**
Median number of cycles (min-max)	3 (1–8)	3 (1–6)
**No. of delayed cycles (%)**	**22 (14.8)**	**9 (6.1)**
**Median dose intensity**	**0.78**	**0.86**

With regard to gemcitabine, 44% of arm A patients received > 85% of the scheduled dose, 54% received 50–84%, and only 3% were given < 50%. In arm B, 76% of patients were administered > 85% of the planned dose, while the remaining 24% received 50–84%.

Docetaxel RDI was ≥ 85% in 59% and 68%, and < 50% in only 5% and 8% of arm A and B patients, respectively. Gemcitabine RDI was ≥ 85% in 33% and 74%, and < 50% in 23% and 0% of arm A and B patients, respectively. Evaluating overall relative dose intensity, ≥ 85% was received by 44%, 84–70% by 21% and 69–50% by 21% of arm A patients. In arm B, ≥ 85% was received by 74%, 84–70% by 5% and 69–50% by 21% of patients.

All 77 patients were assessable for treatment safety. Toxicities observed per patient and per cycle are reported in Table 4. As expected, the most important toxicity was hematological. Arm A patients had higher grade 3 and 4 leukopenia (33.3%) and neutropenia (53.8%) and, and more frequent febrile neutropenia (7.6%) than those in arm B (10.5%, 30.3% and 2.6%, respectively). There were no cases of grade 4 anemia or thrombocytopenia in either arm. Non hematological adverse events were rare and mild: grade 4 toxicity was observed in two arm A patients (1 diarrhea, 1 bowel occlusion) and in three arm B patients (1 cardiotoxicity, 1 pulmonary edema, 1 hepatotoxicity). One case of interstitial pneumonitiis occurred in arm B. There were no toxic deaths.

**Table 4:** Toxicity of docetaxel and gemcitabine combinations per patient

	**Arm A** **n = 39**						**Arm B** **n = 38**					
		
**Toxicity grade**	**2**		**3**		**4**		**2**		**3**		**4**	
**Hematological**	**n**	**(%)**	**N**	**(%)**	**n**	**(%)**	**n**	**(%)**	**n**	**(%)**	**n**	**(%)**
Leukopenia	6	(15)	9	(23)	4	(10)	3	(8)	2	(5)	2	(5)
Anemia	12	(31)	1	(3)	0	-	5	(13)	0	-	0	-
Thrombocytopenia	1	(3)	2	(5)	0	-	2	(5)	0	-	0	-
Neutropenia	3	(8)	7	(18)	14*	(36)	6	(16)	4	(11)	7^†^	(8)
**Non hematological**												
												
Hepatotoxicity	4	(10)	3	(8)	0	-	5	(13)	3	(8)	1	(3)
Mucositis	5	(13)	3	(8)	0	-	2	(5)	0	-	0	-
Nausea	3	(8)	3	(8)	0	-	3	(8)	2	(5)	0	-
Vomiting	3	(8)	2	(5)	0		2	(5)	2	(5)	0	-
Diarrhea	7	(18)	3	(8)	1	(3)	5	(13)	0	-	0	-
Asthenia	8	(21)	2	(5)	0	-	1	(3)	2	(5)	0	-
Alopecia	4	(10)	2	(5)	0	-	0	-	3	(8)	0	-
Skin	5	(13)	0	-	0	-	2	(5)	0	-	0	-
Other	8	(21)	3	(8)	1^§^3)	(3)	8	(21)	0	-	2^‡^	(5)

*3 febrile neutropenia; ^§ ^bowel occlusion; ^†^1 febrile neutropenia; ^‡^1 cardiac toxicity, 1 pulmonary edema

## Discussion

Platinum-based combination chemotherapy is currently regarded as the gold standard of care for advanced NSCLC. In a large meta-analysis published by the NSCLC Collaborative Group in 1995, cisplatin-containing therapy for advanced disease was shown to confer an absolute survival gain of 10% at 1 year, and a modest 1.5-month improvement in median survival compared with best supportive care alone [2]. However, treatment options remain limited as a result of the poor activity of cytotoxic agents.

Docetaxel and gemcitabine have non-overlapping toxicities and several combination regimens of these agents have been evaluated in NSCLC. The every-3-week schedules used consist of docetaxel 75–100 mg/m^2^on day 1 or day 8 and gemcitabine 900–1000 mg/m^2 ^on days 1 and 8. Response rates of 26–50% and a median overall survival of 7–13 months have been reported in phase II trials [19-23].

In the present study, we evaluated 2 different combinations of these two agents in patients with non pretreated metastatic disease. The experimental regimen was derived from our *in vitro *investigation and subsequent dose-finding study, the results of which indicated the safety and feasibility of the sequential treatment [5]. The other schedule was taken from a clinical scheme reported in the literature.

Our purpose was to analyze the activity and safety of both schedules and to determine whether the preclinical results would be confirmed in a clinical trial. The response rates for both non-platinum doublets were (20% and 18%) slightly lower than those historically observed among advanced NSCLC patients with previously untreated disease and a good performance status. It must be pointed out that we only recruited stage IV patients, consequently those with the worst prognosis, and that this choice may have influenced the study outcome. Both regimens had an acceptable toxicity profile and the frequency of grade 3/4 toxicities did not preclude treatment administration in an outpatient setting.

## Conclusion

Our results highlighted acceptable activity and survival outcomes for both the experimental and empirical schedules as first-line treatment. Nevertheless, in agreement with previous studies, platinum-based chemotherapy should currently be considered the reference regimen for the first-line treatment of NSCLC. The docetaxel and gemcitabine combination may be especially useful in patients who have experienced intolerance to platinum, or who may be more susceptible to platinum-related toxicity (e.g. patients with pre-existing renal disease or neurotoxicity). Further studies are now needed to evaluate gemcitabine – docetaxel in combination with emerging molecular agents showing therapeutic potential for advanced NSCLC.

## Abbreviations

NSCLC: non small cell lung cancer; TTP: time to progression; OS: overall survival; CR: complete response; PR: partial response; SD: stable disease; PD: progressive disease; PFS: progression-free survival.

## Competing interests

The authors declare that they have no competing interests.

## Authors' contributions

AP, GLF, CM, LC and DA conceived and designed the study. LC, CD, GO, EP and CM were responsible for patient care and data acquisition. FZ was in charge of quality control and monitoring. MDA and ON were responsible for data management and statistical analyses. AP, GLF and DAM wrote the first draft of the manuscript. WZ carried out the preclinical study. AP, DA and CM critically revised the manuscript for important intellectual content. All authors reviewed and commented on the final manuscript and approved the decision to submit it for publication.
